# Identification of genes associated with complex traits by testing the genetic dissimilarity between individuals

**DOI:** 10.1186/1753-6561-5-S9-S120

**Published:** 2011-11-29

**Authors:** Yan V Sun, Wei Zhao, Kerby A Shedden, Sharon LR Kardia

**Affiliations:** 1 Department of Epidemiology, School of Public Health, Emory University, 1518 Clifton Road NE, Atlanta, GA 30322, USA; 2 Department of Epidemiology, School of Public Health, University of Michigan, 1415 Washington Heights, Ann Arbor, MI 48109-2029, USA; 3 Department of Statistics, University of Michigan, 439 West Hall, 1085 South University Avenue, Ann Arbor, MI 48109-1107, USA

## Abstract

Using the exome sequencing data from 697 unrelated individuals and their simulated disease phenotypes from Genetic Analysis Workshop 17, we develop and apply a gene-based method to identify the relationship between a gene with multiple rare genetic variants and a phenotype. The method is based on the Mantel test, which assesses the correlation between two distance matrices using a permutation procedure. Using up to 100,000 permutations to estimate the statistical significance in 200 replicate data sets, we found that the method had 5.1% type I error at an *α* level of 0.05 and had various power to detect genes with simulated genetic associations. *FLT1* and *KDR* had the most significant correlations with Q1 and were replicated 170 and 24 times, respectively, in 200 simulated data sets using a Bonferroni corrected *p*-value of 0.05 as a threshold. These results suggest that the distance correlation method can be used to identify genotype-phenotype association when multiple rare genetic variants in a gene are involved.

## Background

Genome-wide association studies have successfully identified hundreds of novel genetic loci associated with common diseases; however, only a small portion of the heritability can be explained by these associated common variants. An alternative but not mutually exclusive hypothesis to account for a sizable proportion of genetic susceptibility to common diseases proposes a summation of effects of rare variants in many genes, each conferring an increase in relative risk. In contrast to common variants associated with small effects, rare variants located in a functional region (e.g., exons) are more likely to cause functional effects themselves.

As a result of the low allele frequencies, traditional regression-based methods do not work well with the rare variants derived from the sequencing data. A few methods have been developed to address this challenge by summarizing individual rare variants for association analysis; they are reviewed in a Genetic Analysis Workshop 17 (GAW17) summary paper [1]. For exome sequencing data, a convenient unit of summarizing genetic variants is the gene. GAW17 provides exome sequencing data from the 1000 Genomes Project and simulated phenotypic traits, both binary and quantitative.

In this study, we explore the gene-based analyses to identify genes associated with these traits by summarizing all rare variants within a gene. We develop a gene-based method by testing the correlation between the dissimilarity (measured as pairwise distances between subjects) of the trait and the genotype. We hypothesize that subject pairs that have similar phenotypes will also have similar genotypes within certain genes and conversely that subject pairs with dissimilar phenotypes will have dissimilar phenotypes. Based on the Mantel test [2], we perform a series of analyses to identify genotype-phenotype associations of this type. With up to 100,000 permutations to compute the empirical *p*-values, using this approach, we were able to identify genes that as a whole were associated with the simulated traits after correcting for multiple testing. We also examine the type I error rate and power of the Mantel-based method using the GAW17 simulation answers.

## Methods

### Data

In this study, we use the data set with 697 unrelated individuals provided by GAW17 to conduct the gene-based analysis. The genotypes, sex, and population data of these individuals are from the 1000 Genomes Project [3]. Two hundred replicates of the trait simulation were carried out. The genotypes were held fixed for all 200 simulation replicates. There are 24,487 autosomal SNPs on 3,205 genes available. For each SNP, the name of the SNP, its chromosome and base-pair position, the name of the gene in which it is located, whether the SNP is synonymous or nonsynonymous, and the minor allele and minor allele frequency (MAF) were also provided. Three quantitative traits (Q1, Q2, and Q4) and a binary trait (Affected, coded 0 = no and 1 = yes) were available for each replicate. The Age and Sex variables were fixed across all 200 replicates, and the smoking status covariate, Smoke, varied across the replicates [4].

Among the 24,487 SNPs, 87.2% (21,355) have MAF < 0.05, 74% (18,131) have MAF < 0.01, and 38.5% (9,433) have MAF < 0.0008 (singleton). In terms of the putative function, 13,572 SNPs are nonsynonymous, 10,113 SNPs are synonymous, and 802 SNPs have unknown functional annotation. SNPs with MAF < 0.05 (rare SNPs) were included in this study. A total of 21,355 rare SNPs on 2,881 genes were available for the primary gene-based analysis. The nonsynonymous SNPs with MAF < 0.05 were analyzed separately to understand the effect of the putative function of SNPs. A total of 12,193 nonsynonymous rare SNPs on 2,015 genes were included for the secondary gene-based analysis. Seven hundred ten genes had only one nonsynonymous SNP, and the highest number of nonsynonymous SNPs within a gene was 151.

### Mantel test of correlation between data matrices

The Mantel test is a statistical test of the dependence between the elements of two matrices [2]. Usually, the two matrices contain data from multiple variables obtained on a common sample of subjects. The rows of the two matrices correspond to the subjects in the same order, and the columns contain data on the two sets of variables. For *n* subjects with two variables *X* and *Y*, we first calculate two distance matrices, each with *n* × (*n* − 1)/2 pairwise distances. The Mantel statistic is based on a cross-product term:(1)

where *X* and *Y* are variables measured for the subjects, *n* is the number of subjects in the distance matrices, and *X_ij_* and *Y_ij_* are the pairwise distances between subject *i* and *j* for variables *X* and *Y*. Because the elements of a distance matrix are not independent, it is not straightforward to determine the significance level for the correlation (i.e., Mantel statistic *Z*) between two distance matrices. The Mantel test provides an alternative way to quantify the dependence and provides a significance level that is usually evaluated with a permutation procedure. Mantel’s statistics *Z* are computed for each permuted distance matrix by shuffling the rows and columns. The distribution of *Z*’s is generated by a large number of iterations.

Although the Mantel test was initially developed to identify the space-time clustering in epidemiological data, it has been widely adopted in other fields, such as ecology [5,6]. The Mantel test has also been applied in studies of gene expression profiles and genetics of human diseases [7,8]. Beckmann et al. [8] demonstrated that the Mantel test has better power than the chi-square test for gene mapping using haplotype sharing as a measure of genetic similarity.

### Application of the Mantel test for identifying gene-based correlations

For a given gene, the genetic distance between each pair of subjects (i.e., *X_ij_*) is calculated using the sum of differences of the additive effects on each rare SNP. For a SNP, the distance between two homozygotes (*AA* and *aa*) is 2 and the distance between a homozygote (*AA* or *aa*) and a heterozygote (*Aa*) is 1. The genetic distance on the gene level equals the sum of the genetic distance of individual SNPs. For a gene involving two biallelic loci, *A*/*a* and *B*/*b*, the genetic distance between a pair of individuals ranges from 0 (same genotype) to 4 (*AABB* vs. *aabb*).

We calculate the distance matrices of all rare SNPs (MAF < 0.05) and nonsynonymous SNPs separately for each gene. The phenotypic distance (i.e., *Y_ij_*) equals the absolute difference of the phenotypic values for a pair of individuals (|*Y_i_* − *Y_j_*|). For the quantitative trait Q1, we calculate the phenotypic distances among the unadjusted measurements and their Age, Sex, Smoke, and population stratification adjusted residues. For a binary outcome, the distance between a case subject and a control subject is 1, and the distance among case subjects or among control subjects is 0. Using both the genetic and phenotypic distance matrices, we calculate the Mantel statistic *Z* in Eq. (1) for each gene and a trait.

To estimate the statistical significance of the Mantel statistic *Z*, we first run 500 permutations to compute the empirical *p*-value for each gene with at least one rare variant. For genes with a permutation *p*-value less than 0.002, we rerun the permutation test 100,000 times to obtain a greater precision of the *p*-value as low as 10^−5^. We calculate type I error using quantitative trait Q4, which has no association with the genetic variants, in 200 replicates. We also calculate the power for the nine genes with causal SNPs of quantitative trait Q1 using three significance thresholds (0.05, 0.001, and 0.0001). All statistical analyses were conducted using statistical software R, version 2.10. The Mantel test was implemented in R library ade4 [9].

## Results

Because of the availability of the underlying genetic model of simulation for GAW17, we first examined the type I error and power of our gene-based method. Using Q4, which has no simulated genetic associations, we estimated that the mean of the type I error (*α* level of 0.05) for our Mantel test–based method was 0.051 in 200 replicate data sets. The power analysis of this method is summarized in Table 1. By combining the nonsynonymous SNPs with MAF < 0.05 of a gene, we were able to calculate the true-positive rate of the nine genes with simulated genetic associations. Each gene included at least one causal SNP with various effect sizes. At an *α* level of 0.05, three genes were identified with reasonable power: 100% power for *FLT1*, 96% power for *KDR*, and 78% power for *VEGFC*. Considering a large number of tested genes in this study, we also examine the power at *α* levels of 0.001 and 0.0001. We have power to detect only *FLT1* using these lower thresholds (Table 1).

**Table 1:** Power of identifying nine genes with simulated genetic association

Gene	Power			Number of causal SNPs/total SNPs		
	
	*α* = 0.05	*α* = 0.001	*α* = 0.0001	All SNPs	MAF < 5%	MAF ≥ 5%
*ARNT*	0.255	0	0	5/18	5/17	0/1
*ELAVL4*	0.01	0	0	2/10	2/8	0/2
*FLT1*	1	0.97	0.95	11/35	10/32	1/3
*FLT4*	0.135	0.01	0	2/10	2/10	0/0
*HIF1A*	0.17	0	0	4/8	4/8	0/0
*HIF3A*	0.09	0	0	3/21	3/17	0/4
*KDR*	0.955	0.525	0.245	10/16	9/15	1/1
*VEGFA*	0.225	0.005	0.005	1/6	1/6	0/0
*VEGFC*	0.775	0	0	1/1	1/1	0/0

Following the procedure described in the Methods section, we next tested the association between the genetic dissimilarity of each gene and the phenotypic dissimilarity represented by the distance matrices of Q1 and Affected. After excluding all common SNPs, we conducted the gene-based analysis using either all SNPs or only nonsynonymous SNPs for each trait, using up to 100,000 permutation tests. For the quantitative trait Q1, we also considered models with and without adjustment of covariates (Age, Sex, Smoke, and population stratification).

The most significant association results (significantly associated with the outcome more than 10 times out of 200 replicates, i.e., 5%) of the four models, three for Q1 and one for Affected, are summarized in Table 2. Using a stringent significance threshold of a Bonferroni-corrected *p*-value of 0.05 (empirical *p*-values of 1.74 × 10^−5^ for SNPs with MAF < 0.05 and 2.48 × 10^−5^ for nonsynonymous SNPs with MAF < 0.05), we identified four genes associated with Q1 and one gene associated with Affected in more than 10 of the 200 simulated data sets. Without any adjustment of covariates, *FLT1* (32 rare SNPs), *PIK3C3* (7 rare SNPs), *KDR* (15 rare SNPs), and *PRR4* (17 rare SNPs) were significantly associated with Q1 49, 13, 12, and 11 times, respectively, out of 200 simulation data sets. With adjustment of Age, Sex, Smoke, and the first two principal components of all SNPs (representing population stratification among subjects), *FLT1* was significant 39 times. When we tested the gene-based association by considering only nonsynonymous SNPs, we found that *FLT1* (19 nonsynonymous SNPs) and *KDR* (10 nonsynonymous SNPs) were significant 170 (85%) and 24 (12%) times, respectively. For the binary outcome Affected, no gene was significant more than 10 times among 200 simulated data sets using all SNPs with MAF < 0.05. The most significant genes, *MAP3K12* (17 rare SNPs) and *PIK3C2B* (62 rare SNPs), were significant 10 and 5 times, respectively. When we restricted the analysis to only nonsynonymous SNPs, we found that *FLT1* (19 nonsynonymous SNPs) was significant 13 times.

**Table 2:** Most significant genes correlated with Q1 and Affected

Trait	Gene	Chromosome	Gene start (bp)	Gene end (bp)	Gene length (bp)	Number of SNPs	Number of significant tests^a^
Q1 (SNPs with MAF < 0.05)	*FLT1*	13	27774389	27967265	192877	32	49
	*PIK3C3*	18	37829928	37789197	126250	7	13
	*KDR*	4	55639406	55686519	47114	15	12
	*PRR4*	12	10889715	11215480	325766	17	11
Q1 (SNPs with MAF < 0.05)^b^	*FLT1*	13	27774389	27967265	192877	32	39
Q1 (nonsynonymous SNPs with MAF < 0.05)^b^	*FLT1*	13	27774389	27967265	192877	19	170
	*KDR*	4	55639406	55686519	47114	10	24
Affected (nonsynonymous SNPs with MAF < 0.05)	*FLT1*	13	27774389	27967265	192877	19	13

^a^ Number of significant tests out of 200 simulated data sets. The threshold of statistical significance is a Bonferroni-corrected p-value of 0.05 (1.74 × 10^−5^ for SNPs with MAF < 0.05 and 2.48 × 10^−5^ for nonsynonymous SNPs with MAF < 0.05).

^b^ Adjusted for Age, Sex, Smoke, and first two principal components.

## Discussion

The exome sequencing data measure a large number of rare variants in which a subset may be jointly associated with disease phenotypes. The gene-based analyses can divide these rare variants into genes as a unit and imply a relationship between a gene and a phenotype. In this study, we developed a gene-based Mantel test to assess the correlation between a phenotype and all rare variants of a gene. The Mantel test is capable of evaluating the relationship between the distance matrices of phenotype and genotype using a permutation process. Although this method can be applied to summarize any type of genetic variant, including common variants within a genomic region, in this study we focused on identifying rare variants, which may not have sufficient power to be detected individually. We applied this method to the GAW17 unrelated individuals data and identified genes with rare SNPs that were significantly correlated with one quantitative trait and the binary trait. Using the 200 simulated data sets and comparing with the underlying genetic models, we found that this method had an expected type I error rate and that the power to detect gene-level association of rare variants depended on the number of causal rare variants and their effect size. When an appropriate subset of SNPs, such as SNPs with low MAF and nonsynonymous SNPs, and an appropriate adjustment model were selected, the method had improved performance in detecting the associated genes. Using the stringent Bonferroni correction for multiple testing implemented in this study, we were not surprised by the number of false negatives. Using a less stringent correction for multiple testing (e.g., false discovery rate *q*-value) may help to reduce the false-negative rate.

The method we implemented here provides an alternative way to test the relationship between a phenotype and all genetic variants located within a gene. The Mantel test is designed to identify not only the association between predictors and outcome but also the clustering of the events within the predictor-outcome space [2]. In addition, this framework is flexible so that any set of rare variants can be combined to test their joint correlation with a phenotype. For example, we can test the hypothesis involving all genes in a known pathway, a biological network, or any set of genes grouped by a proposed mechanism. Another advantage of our method is its capability of handling different genetic models. Here, we coded the genotypes using an additive effect model and calculated the distance matrix of a gene. Similarly, we could have tested the correlation of the dominant or recessive effects by modifying the coding of the genotypes and calculating the new genetic distance matrices.

The Mantel test assesses a global-level relationship (i.e., correlation) for all variables. It cannot select the most influential independent variables. In this study of exome sequencing data, we could make inferences on the gene level but were limited in how much we could narrow down the list of causal variants under the framework of the Mantel test. Combining our gene-based approach with variable selection or ordination methods may facilitate the process of uncovering causal variants of human disease. In the analyses for identifying genes with multiple rare variants jointly correlated with disease traits using the exome sequencing data, we believe that the Mantel test can play an important role in understanding the complicated genetic effects of rare variants. Further developments are needed to extend the utility of the Mantel test in whole-exome sequencing data and for fine mapping of causal variants.

## Competing interests

The authors declare that there are no competing interests.

## Authors’ contributions

YVS conceived of the study, drafted the manuscript and performed the statistical analysis. WZ participated in the design of the study and performed the statistical analysis. KAS and SLRK participated in its design and helped to draft the manuscript. All authors read and approved the final manuscript.
